# Supplementary material for: Surface Microbiome Profiling of Dental Elevators Using Third‐Generation Sequencing: Implications for Infection Control in Dental Practice
Source: Microbiologyopen. 2025 Dec 8;14(6):e70178. doi: 10.1002/mbo3.70178 (PMC12685768; doi:10.1002/mbo3.70178)
Supplement: Supplementary file 1 — customer_backup. [file MBO3-14-e70178-s001.zip › customer_backup/customer_backup/taxa_summary/krona/samples/M17.Krona.html]

Javascript must be enabled to view this page.

members
magnitude
magnitudeUnassigned

M17.krona

55010

54999

81

81

81

81

81

27

16

22

2

14

10880

11

11

1

1

1

2

2

2

8

8

1

7

111

111

111

111

111

6022

140

140

17

17

123

93

1

7

2

6

2

1

11

6

6

6

6

53

53

53

53

1

1

1

1

49

49

49

5

7

11

3

23

18

18

2

2

1

1

2

2

5

5

7

7

1

1

680

168

3

3

160

160

2

2

2

2

1

1

512

29

10

19

445

9

10

2

155

29

10

16

23

25

6

2

83

8

9

2

50

6

5

5

33

2

31

11

11

11

5

6

28

28

28

3

17

8

73

8

1

1

7

7

4

4

1

3

61

55

2

53

4

4

2

2

277

137

29

29

108

108

19

2

2

17

17

121

2

2

25

25

94

94

38

38

6

3

3

19

3

1

15

2

2

1

1

2

2

3

2

1

5

5

4638

14

4

4

6

6

3

3

1

1

4137

1

1

491

491

4

4

642

642

2999

4

1960

46

826

163

375

346

342

4

29

29

84

4

4

4

4

8

8

4

4

38

2

36

10

9

1

16

16

28

27

27

1

1

10

2

2

2

8

5

5

3

3

2709

1381

12

12

12

652

439

101

6

13

137

129

53

20

20

71

71

92

62

15

15

30

30

717

88

88

624

624

5

5

1328

600

600

24

576

728

728

728

1972

106

103

3

3

67

67

1

1

8

8

12

12

12

12

3

3

3

215

44

44

44

25

25

25

146

146

146

465

22

22

22

24

24

24

60

60

60

14

14

14

303

303

303

42

42

42

2

2

2

2

1184

119

111

111

8

4

4

324

324

8

316

384

384

384

357

27

27

313

313

17

17

55

55

55

55

8

44

3

4

4

4

4

1

1

2

1

1

1

1

1043

1043

1043

1043

1043

1043

42

42

42

42

2

2

3

3

12

12

25

24

1

27

27

27

8

8

8

3

3

3

4

4

4

12

12

12

81

4

4

4

4

4

21

21

6

6

6

15

15

15

56

56

56

56

56

140

13

1

1

1

1

12

12

12

12

127

125

125

125

125

2

1

1

1

1

1

1

19

19

19

19

19

14

5

162

43

43

43

1

1

33

8

2

23

5

5

4

4

2

2

2

2

1

1

2

2

2

2

2

66

66

6

6

6

60

60

4

42

14

20

20

20

20

20

29

29

29

29

29

35

35

35

35

35

35

641

206

5

5

5

5

201

80

56

56

6

6

18

18

121

44

44

1

1

76

76

136

25

25

25

1

20

4

3

3

3

3

108

108

38

16

22

25

25

27

10

17

18

5

13

26

22

22

22

22

4

4

4

4

80

80

80

4

4

76

43

3

30

8

8

8

8

8

185

185

17

17

17

167

167

167

1

1

1

2216

16

3

3

3

3

13

13

13

13

90

10

10

10

10

57

26

26

26

31

31

31

7

7

7

7

7

7

7

7

9

9

9

9

17

12

12

12

12

5

3

3

3

2

2

2

2093

2093

1503

1503

1503

25

25

25

60

60

60

276

185

185

7

7

77

77

7

7

29

29

29

45

45

45

155

155

155

10

10

6

6

6

6

4

4

4

4

21

21

21

17

17

17

4

4

4

722

14

14

14

14

14

254

254

254

17

17

121

121

103

10

93

13

5

8

454

454

454

341

2

190

1

148

100

100

13

13

626

173

173

173

173

173

25

25

25

25

25

428

133

133

1

1

132

48

84

1

1

1

1

259

57

6

6

6

6

45

15

30

192

7

7

185

185

10

10

10

35

35

4

4

31

31

111

111

111

24

24

24

87

40

7

33

47

15

5

5

4

18

183

127

17

17

9

9

8

5

3

5

5

5

5

105

40

40

40

34

34

34

9

9

9

11

11

11

11

11

11

56

32

32

9

9

23

23

24

24

24

4

20

29294

20319

173

173

98

5

93

75

75

593

35

35

35

456

6

6

45

24

21

30

30

375

1

364

10

102

102

102

21

21

21

21

40

40

29

29

11

11

18

13

13

13

5

5

5

7565

7565

253

28

9

82

6

128

94

94

30

10

2

18

1019

659

124

236

197

197

2

2

191

7

184

5779

2

116

4

1

2

21

21

474

79

21

1546

1295

1851

15

10

20

288

5

2

6

16

16

16

16

3246

222

4

4

11

11

20

19

1

187

54

20

113

27

27

27

8

8

8

7

7

7

323

151

20

5

86

40

48

48

124

124

7

7

7

109

5

5

6

4

2

6

6

92

8

46

38

71

52

30

22

19

19

991

9

9

41

41

185

185

259

259

372

17

97

197

2

5

54

14

14

18

18

3

3

90

13

40

14

23

277

16

16

4

4

13

13

40

40

33

33

171

47

124

103

8

8

70

70

25

25

1

1

1

1100

352

352

10

9

1

4

4

536

3

2

3

130

61

106

103

37

4

74

13

5

5

26

26

24

5

19

1

1

142

142

15

15

15

15

307

307

161

14

92

55

46

4

42

19

15

4

22

22

59

54

5

17

17

17

17

27

27

3

3

24

22

2

2

2

2

2

135

135

135

133

2

1

1

1

1

6498

6498

56

56

41

41

6218

6218

145

94

51

22

1

10

11

12

5

7

4

4

8

8

8

8

375

40

40

40

241

241

241

7

7

7

4

4

4

83

83

83

1262

1262

5

5

58

58

23

21

2

25

20

5

12

12

1028

20

463

545

111

111

8975

8

8

8

8

7

7

7

7

33

1

1

1

3

3

3

23

3

3

10

10

10

10

2

2

2

4

2

2

1

1

1

1

5204

24

24

24

7

1

1

6

6

23

9

9

14

14

37

37

37

2702

823

823

699

53

1

45

600

1180

13

81

54

25

5

68

11

30

9

34

45

56

691

58

103

5

5

59

59

22

22

17

17

9

9

9

96

8

8

70

3

67

18

11

7

61

26

26

35

35

2006

6

6

17

17

30

30

102

102

16

16

165

148

17

209

112

96

1

808

111

697

142

142

19

19

109

109

383

383

134

134

134

2

2

2

147

38

29

28

1

9

9

109

105

23

82

4

4

68

68

18

12

1

5

9

9

31

4

27

10

10

1

1

1

1

34

20

20

20

14

14

14

16

16

16

9

1

6

83

83

83

83

3000

708

164

164

38

38

506

506

2292

18

18

16

16

38

38

24

14

10

2196

620

6

759

112

43

25

8

37

586

8

8

8

8

13

13

13

13

17

17

17

17

336

1

1

1

298

298

295

3

13

2

2

11

7

4

5

5

5

19

8

5

3

6

6

5

5

17

17

17

17

17

17

541

399

399

399

26

26

55

55

13

13

69

69

116

18

98

120

16

78

26

142

5

5

5

5

20

11

11

11

9

9

9

55

24

8

5

3

16

11

5

30

4

1

3

26

11

15

1

1

1

29

29

18

18

11

11

33

33

33

33

84

84

84

84

84

32

3

49

4755

1015

1

1

1

1

2

2

2

2

9

9

9

9

731

731

1

1

13

2

11

7

3

3

1

61

15

46

20

12

8

14

5

8

1

5

2

2

1

14

12

2

23

23

30

1

29

59

59

1

1

3

1

2

1

1

2

2

52

17

34

1

1

1

74

74

1

1

37

3

34

310

182

6

33

66

6

3

1

1

12

2

2

72

14

12

12

2

1

1

29

3

3

1

1

1

1

24

23

1

29

29

29

33

33

6

6

27

26

1

12

2

2

2

10

10

10

148

116

5

1

1

3

44

44

1

1

10

1

9

1

1

55

55

9

9

9

9

6

1

5

2

1

1

1

1

14

14

14

7

7

7

7

3682

60

60

1

1

59

4

55

463

463

417

72

102

137

2

9

6

4

19

18

13

5

30

18

18

28

16

12

1

1

1

1

1744

13

13

11

2

170

168

1

21

3

38

8

94

3

2

2

3

3

2

1

1558

156

156

66

66

10

5

1

4

1016

1

313

702

88

1

62

5

20

16

16

73

56

1

16

9

9

53

53

9

1

8

2

2

60

55

5

1249

903

13

13

9

9

881

161

536

11

140

9

9

9

6

346

233

233

32

5

27

45

45

24

24

6

5

1

6

6

6

6

6

2

4

1

1

1

1

158

37

34

34

1

1

1

1

1

1

121

25

23

2

12

12

58

58

5

5

1

1

3

3

11

11

4

2

2

1

1

1

1

6

6

6

6

2

2

2

52

52

52

52

52

177

41

2

2

2

2

21

21

21

21

18

8

8

8

10

10

10

7

7

7

7

7

47

11

11

11

11

6

6

3

3

1

1

2

2

6

6

6

6

16

16

16

16

8

8

8

8

82

82

82

82

82

16

16

16

16

16

16

10

10

10

10

10

10

207

207

207

207

207

107

100

406

322

5

5

5

5

4

4

4

4

313

307

307

307

6

6

6

84

84

84

26

26

34

34

24

24

2400

2387

1458

1

1

1

1369

10

10

29

29

6

6

8

8

573

13

23

1

476

12

23

25

257

145

79

33

34

12

22

128

128

185

68

114

3

139

139

88

88

4

12

72

161

11

2

2

2

2

7

7

8

8

8

142

142

142

279

1

1

1

12

12

12

146

1

1

6

6

41

41

98

98

13

2

2

11

6

2

3

5

4

4

1

1

2

2

1

1

46

46

6

18

8

2

4

3

1

2

2

15

1

1

1

1

13

3

1

9

12

12

12

27

3

3

17

17

3

3

2

2

1

1

1

1

26

6

6

6

20

6

6

14

14

463

40

14

14

26

11

15

10

10

10

31

3

3

22

5

17

6

6

62

58

58

4

4

320

2

2

153

4

20

88

24

17

4

4

3

3

158

19

11

12

82

12

7

5

4

6

1

1

1

1

1

11

11

7

7

7

4

4

4

1

1

1

1

1

12

12

12

2

2

2

10

10

10

36

36

36

36

36

36

8

8

8

8

8

8

8

3

2

2

2

2

1

1

1

1

1

1

1

1

1

1
